# The Texas State Society of Social Hygiene

**Published:** 1913-08

**Authors:** 


### Department of Eugenics.

Conducted by Dr. Malone Duggan and Dr. Theo. Y. Hull.

### THE TEXAS STATE SOCIETY OF SOCIAL HYGIENE.

Headquarters: San Antonio, Texas.

Dr. Malone Duggan, President, San Antonio.
Dr. F. E. Daniel, 1st Vice President, Austin.
Prof. A. Caswell Ellis, 2d Vice President, Austin.
Mrs. Eli Hertzberg, 3d Vice President, San Antonio.
Dr. Theo. Y. Hull, Secretary, San Antonio.
Mr. W. L. Evans, Treasurer, San Antonio.

EXECUTIVE COMMITTEE.

| | | |
|---|---|---|
| Dr. Malone Duggan, Chm. | Hon. J. C. Willacy. | Dr. W. A. King. |
| Dr. Theo. Y. Hull, Sec'y. | Mr. W. L. Evans. | Dr. J. W. Torbett. |
| Dr. W. F. McCaleb. | Dr. Frank D. Boyd. | Dr. F. C. Walsh. |
| Hon. C. H. Jenkins. | Dr. J. R. Nichols. | Dr. Joe Reuss. |
| Rev. A. W. S. Garden. | Dr. J. M. McCutchan. | Dr. Frank Paschal. |

Do you know that in every ten seconds the life of an infant flashes out; that every tenth child dies before it is a week old, and that every fourth one perishes before it is a year old? That this is true is the result of ignorance. Ignorance not only of how to rear the child, but of the more important fact of what constitutes a healthy organism. When will we ever learn the lesson that the greatest humanity lies in the right of the child to be well born?

A Pertinate Observation.—Apropos to the above statement are the remarks made by Dr. Newell Dwight Hillis, "That a State that requires two years' study for a man to practice pharmacy, four years for law and six years for medicine should certainly require as rigid an examination for a man and woman about to establish an American home." When will we realize the great responsibility of parenthood? When will the State exercise its rightful function to safeguard its future interest deliberately and judiciously by regulating reproduction?

The correct attitude is displayed in the example of Dean Summer when he gave out notices that no one need come to him to be married unless he be free from communicable disease. This is the best possible way to educate the public to the full realization of the morality of marriage. Next to this in point of effectiveness would be a law on our statute books compelling a health certificate before the issuing of license to marry and to make it a felonious offense for any person to infect another with a venereal disease.

Dense ignorance and lack of proper appreciation of his important position in society was recently displayed by a well known minister in one of our largest cities when he publicly united in marriage two deaf mutes, thereby legalizing them to commit a crime against nature in bringing into the world children handicapped by either mental or physical deformity, the only crime that nature knows. This minister, it is true, might excuse himself (if he had any misgivings about the matter), on the grounds that he was acting in the interest of good morals and in recognition of the inalienable right of man to exercise his prerogative in the pursuit of happiness, and that if any crime was committed the burden fell on the State in not recognizing its responsibility and providing in such instances the sterilization (not castration) of both parties, thereby not in the least interfering with "inalienable rights" and at the same time prevent the crime of producing other unfortunate deaf mutes.

Starting Right.—What great significance is expressed in these words, "starting right!" Would you not feel more comfortable when your daughter married if you had procured a health certificate from her intended husband, showing that he was physically and mentally clean? Have you not the same interest in every other good woman in the land and in the perpetuity of society? Alas! When will we distinguish between lust and love? Between mere gratification of physical desire and the sweet companionship of intellectual attraction that satisfies the soul?

Too late might be aptly applied to the father in Brieux's play, "Damaged Goods," when he proposed to take the law into his own hands, since the law provides no protection from the man who marries an innocent, confiding woman, and befouls her with his debauchery. He cries: "When I think of that man and his infamous conduct, I can not control myself! My daughter! A girl of 22!" But the doctor replies: "Are you sure you have the right to be so inflexible? Was it not within your power to spare your daughter this misery? When the marriage was proposed, you made inquiries concerning your future son-in-law's income. You satisfied yourself as to his standing, but you omitted the most important point of all. You made no inquiry concerning his health! You daughter might well ask you: "Who are a man and a father, and ought to know these things?" "Why did you not take as much trouble about her health as about her fortune?"

Seed Begins to Take Root.—The following from the *Chicago Inter-Ocean* indicates how the agitation for proper sex instruction is beginning, at last, to take practical form. The example of the

Chicago schools will set a wholesome example for the entire country: "The Chicago board of education has reached the wise decision to have sex hygiene taught in the high schools. This will be done from the beginning of the next school year."

MUCH OF THE VICE prevalent in the world is the result of inexcusable ignorance. Certain knowledge comes to all young people, and it should come from proper sources. Otherwise it will come from improper sources, tainted with salacious and vicious suggestion, appealing to the love for the mysterious that is innate in all young people.

There being no restraint upon the acquisition of such knowledge, vice naturally follows. The number of young lives that have been ruined through a false and strange modesty that has been set up as standard in our educational system is incalculable.

Young people should be taught, in the usual class room way, of the origin of man, so that no mystery may attach to it. The naturalness of the sex relations, the necessity for their maintenance upon a plane of purity and decency, should be emphasized. The youth of the nation should be taught to reverence, preserve and conserve their bodies and their health.

If sex hygiene is not taught in the schools by proper teachers, these things will be learned from the alleys and the dark places, instead of in the bright light of science and wholesome teaching.

